# Hypoxemic Bronchiolitis Related to Major Histocompatibility Class II Deficiency

**DOI:** 10.1155/2013/315073

**Published:** 2013-08-24

**Authors:** S. Hammami, H. Besbès, S. Hadded, K. Lajmi, L. Ghédira, Ch. B. Meriem, M. N. Guediche

**Affiliations:** Paediatric Department, Fattouma Bourguiba Hospital and University of Monastir, Faculty of Medicine, 5000 Monastir, Tunisia

## Abstract

Major histocompatibility complex class II expression deficiency is an autosomal recessive primary combined immunodeficiency. The prevalence of this deficiency is the highest in Mediterranean areas, especially North Africa. Early diagnosis is essential due to high mortality in the first 2 years of life. Prognosis is very poor when bone marrow transplantation cannot be performed. We report the case of an infant with major histocompatibility complex class II expression deficiency revealed by hypoxemic bronchiolitis due to *Pneumocystis jiroveci*.

## 1. Introduction

Major histocompatibility complex (MHC) class II deficiency is a rare inherited primary combined immunodeficiency syndrome characterized by defective expression of HLA class II antigens [1]. The majority of cases reported are from North Africa. Early diagnosis is essential due to high mortality in the first 2 years of life because of high susceptibility to a broad range of viral, bacterial, fungal, and protozoan infections [2, 3]. We report a new case of this disease revealed by hypoxemic bronchiolitis.

## 2. Case Report

**Figure 1:**
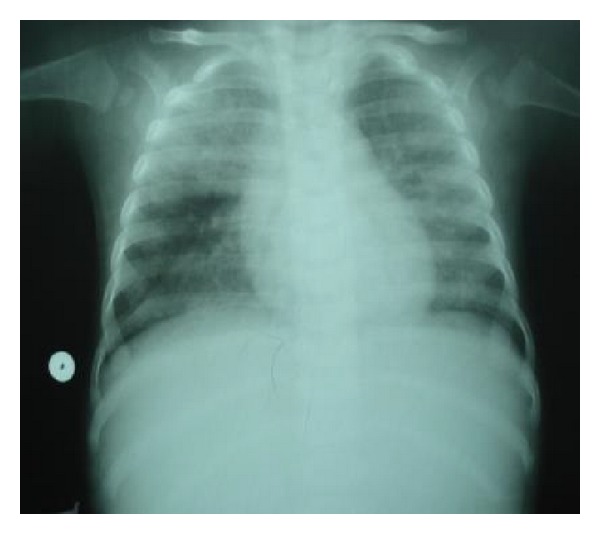
Initial chest X-ray: bilateral alveolointerstitial infiltrate.

**Figure 2:**
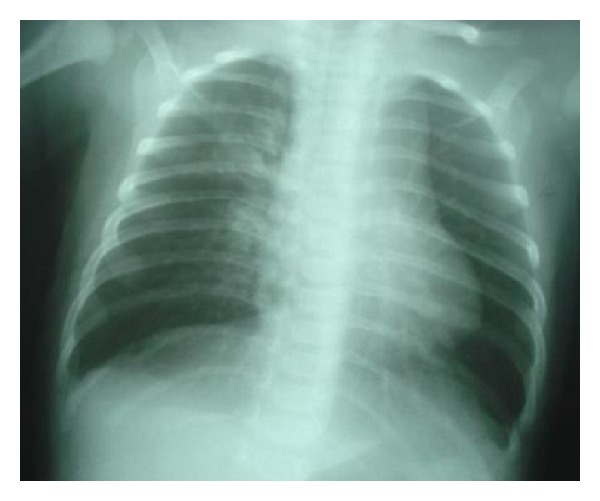
Control chest X-ray: normal.

A 6-month-old infant, of healthy consanguineous parents, presented to our pediatric unit on the January 2, 2012, for fever with cough. The infant had one month's history of polypnoea and cough. He was born at term, weighing 3500 g, with Apgar scores of 9 and 10 at 1 and 5 minutes, respectively. He received breast-feeding for 4 months. Infant had normal psychomotor development. He weighed 4500 g, his height was 60 cm, and temperature was at 38.5°C. Physical examination noted polypnoea, tachycardia without signs of heart failure, and perioral cyanosis with oxygen saturation at 78%. Complete blood cell showed moderate hypochromic microcytic aregenerative anemia and leukocytosis. Iron investigation showed low ferritinemia, compatible with iron deficiency. Inflammatory markers were moderately increased. A chest X-ray revealed bilateral alveolointerstitial infiltrate (Figure 1). Viral serology particularly human immunodeficiency virus was negative. Test for syncytial respiratory virus was negative. Laboratory tests, including renal function, serum electrolytes, and liver enzymes were normal. Blood culture was also negative. Chest computed tomography noted bilateral interstitial infiltrate. Infant received broad-spectrum antibiotic and oxygen. Initial clinical course was unfavorable with persistence of oxygen dependence. *Pneumocystis jiroveci* pneumonitis was evoked. Infant received trimethoprim and sulfamethoxazole (TMP/SMX) at 100 mg/weight/day. Evolution was favorable. Control chest X-ray was normal, and infant was weaned of oxygen (Figure 2). Microbiologic tests revealed an infection with *Pneumocystis jiroveci. *An immunodeficiency disease was suspected based on lingering symptoms associated with poor weight gain. Immunologic investigations allowed us to make the diagnosis of MHC class II deficiency. Infant received antibiotic prophylaxis and monthly immunoglobulin treatment. Unfortunately, infant did not have an HLA-identical donor. Evolution was characterized by appearance of other recurrent infections (respiratory and gastrointestinal tracts). Infant died at age of 18 months from severe denutrition and septicemia.

## 3. Discussion

Major histocompatibility complex class II (MHC-II) expression deficiency is a rare combined immunodeficiency leading to the impairment of the cellular and humoral immune responses [4]. It is an autosomal recessive disease caused by the defective expression of human leucocyte antigen (HLA) class II molecules due to mutated transacting proteins. MHC locus itself is not affected. Four genes have been identified to encode regulatory factors controlling transcription of MHC class II genes: CIITA, RFXANK, RFX5, and RFXAP. The mutations in the RFXANK gene are the most frequent and account for more than two-thirds of all reported cases [5]. In classical presentation, T and B cells are within the normal range, lymphopenia is moderate, and the lymphocyte proliferation in response to stimulation with mitogens is normal. The patient's T cells responded normally to alloantigenic stimulation and also had the capacity to develop antigen-specific cytotoxic functions. However, the T cells were completely naive to soluble protein antigens. The lack of MHC class II expression results in a severe defect in both humoral and cellular immune responses to foreign antigens. Patients have generally severe CD4 T-cell lymphopenia, and hypogammaglobulinemia and lack Ag-specific responses resulting in chronic diarrhea, recurrent viral, parasitic and bacterial infections, and failure to thrive [6]. Viral infections such as cytomegalovirus, enterovirus, adenovirus, and herpes simplex virus were the most common pathogens and the predominant cause of death [7]. Usually, first clinical signs of infections begin in the first year of life. Our patient developed pulmonary infection at the age of six months. Around this age, *Pneumocystis jiroveci* infection is indeed a classical mode of revelation of congenital severe cellular immune deficiency. Patients suffering from MHC-II expression deficiency died at a mean age of 4 years, often of bacterial or viral infections and severe denutrition [8]. Our patient died at age of 18 months of severe denutrition and gastrointestinal infection. However, MHC-II expression deficiency is quite common in North Africa [9–11]. A majority of affected patients belong to consanguineous families particularly from the Maghreb. Careful clinical and biological investigations should allow the discrimination of MHC class II deficiency from other diseases presenting with the same presentation at this age, such as severe combined immune deficiency and HIV infection. At present the only curative therapy is allogenic hematopoietic stem cell transplantation [4, 12]. Prognosis is very poor when bone marrow transplantation cannot be performed. Despite this intensive care, overall cure rate is lower than that in other immunodeficiencies. This reduced survival is well correlated with an increased incidence of acute graft-versus-host disease and preexisting viral infections. Particularly, transplantation of cord blood stem cells may be an effective source of hematopoietic stem cells for patients with immunodeficiency disorders including diseases with a high rate of graft failure [13, 14].

## 4. Conclusion

Report of this new case of major histocompatibility complex class II expression deficiency confirms the frequency of this disease among the North African population. In Maghrebian settings, pediatricians should definitely consider this diagnosis in the presence of an early onset of severe and recurrent infections of the respiratory and intestinal tracts, with a failure to thrive.
